# Electron microscopy of Chaetomium pom152 shows the assembly of ten-bead string

**DOI:** 10.1038/s41421-018-0057-7

**Published:** 2018-09-18

**Authors:** Qi Hao, Boyue Zhang, Kangning Yuan, Hang Shi, Günter Blobel

**Affiliations:** 1 Laboratory of Cell Biology, Howard Hughes Medical Institute, The Rockefeller University, 1230 York Ave., New York, NY 10065 USA; 2 Beijing Advanced Innovation Center for Structural Biology, School of Life Sciences, Tsinghua University, Beijing, 100084 China; 3 Present Address: Calico Life Sciences, 1170 Veterans Blvd, South San Francisco, CA 94080 USA

Dear Editor,

A belt around the waistline of pore membrane (pom) of the nuclear envelope has been detected with electron microscopy^1^. However, its molecular identity, architecture, and function remained unclear. Of the three distinct integral membrane proteins populating the nuclear pore membrane, only pom152 (of yeasts) or gp210 (of multi-cellular organisms) contain a sufficiently large ‘*trans*’ domain (over 100 kDa) that could form such a belt (Fig. 1a and Supplementary Fig. S1).

**Fig. 1:**
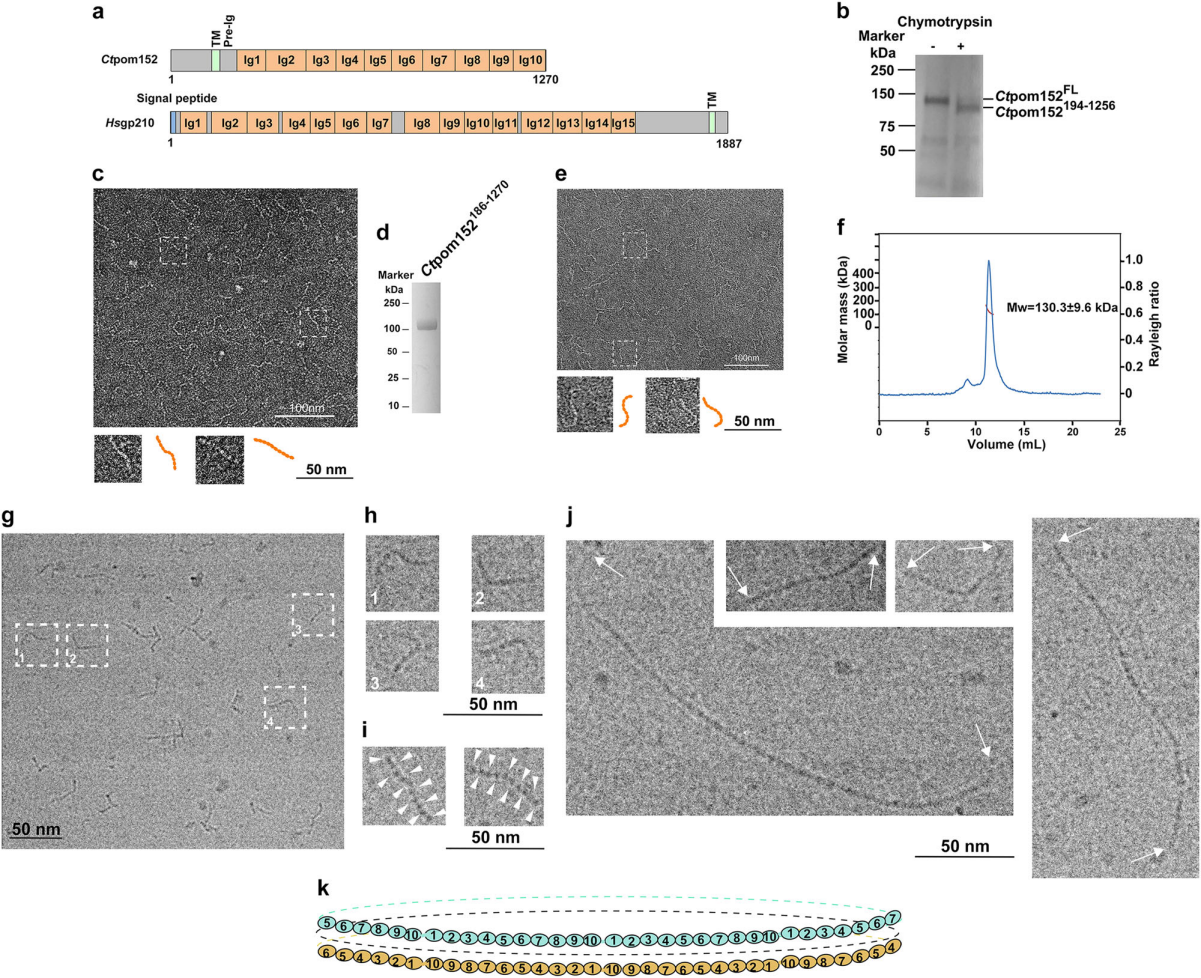
Electron microscopy of *Ct*pom152 reveals the assembly of beaded strings. **a** Domain architecture of *Ct*pom152 and *Hs*gp210. Regions without a predicted fold are indicated in gray; Ig, immunoglobulin (Ig)-like fold; TM, transmembrane segment; pre-Ig, the conserved segment between TM and Ig1. See Supplementary Figs. S1, S2 for details. **b** Limited chymotryptic digestion of full-length (FL) recombinant *Ct*pom152. **c** Negative-stain EM of chymotryptic fragment showed beaded and flexible strings of ~40 nm in length (37 ± 4 nm, number of particles: *N* = 30); two of the beaded strings (two dashed boxes) are shown enlarged (orange, bottom inserts); up to ten beads are discernible. **d** SDS-PAGE and Coomassie Blue staining of purified recombinant *Ct*pom152^186-1270^. **e** Like **c**, negative-stain EM of recombinant *Ct*pom152^186-1270^ also showed beaded string structures (measures 40 ± 5 nm, *N* = 30). **f** SEC-MALS indicates that *Ct*pom152^186-1270^ is a monomer. **g**–**j** Cryo-electron micrograph of *Ct*pom152^FL^. **g** Single particle showed flexible beaded strings (measures 44 ± 3 nm, *N* = 30), as seen in negative-stain EM (**c**, **e**) and four selected particles (orange with dashed box) are enlarged (**h**). **i** Two selected particles with high contrast showing ten beads (indicated by arrow heads) and the large structural variations. **j** *Ct*pom152^FL^ polymerizes into long continuous strings with no punctuation marks. Particles represent the polymer formed by seven (bottom left), two (top), and five copies (right). **k** We speculate that (1) eight *trans* region of pom152 head-to-tail connect into a flexible ring and (2) two anti-parallel, stacked rings form above (cyan, omitted regions represented by dashed line), below (orange, omitted regions represented by dashed line), and mid-plane (black dashed line)

Using the Phyre2 program^2^ to predict tertiary structures of yeast pom152 and its multi-cellular ortholog, gp210, we first identified ten and fifteen, respectively, immunoglobulin-like (Ig-like) folds which extended over much of these proteins’ large *trans* domain (Fig. 1a, Supplementary Figs. S2 and S3). We next expressed full-length (FL) pom152 (*Ct*pom152^FL^) of the thermophilic yeast *Chaetomium thermophilum* in insect cells and purified *Ct*pom152^FL^ with detergent extract. Incubation of purified *Ct*pom152^FL^ with chymotrypsin revealed a large stable fragment (Fig. 1b), which we identified by mass spectrometry as *Ct*pom152^194-1256^ (Fig. 1b). This fragment represents most of the *Ct*pom152 *trans* domain, starting ~40 residues downstream of its transmembrane helix (TM) and having lost 14 of its C-terminal residues (Fig. 1a and Supplementary Fig. S1).

The purified *Ct*pom152^194-1256^ (detergent-free) was examined by negative-stain EM using uranyl acetate. The electron micrographs showed beaded strings of 37 ± 4 nm, with each bead measuring ~4 nm in length and ~2 nm in width (Fig. 1c) with up to ten beads per string, consistent with our structural predictions. On the grid, the beaded strings assumed various shapes, indicating a large degree of flexibility. To further improve the purity and quality, we expressed a new construct *Ct*pom152^186-1270^ in insect cells. *Ct*pom152^186-1270^ lacks the upstream transmembrane segment and thus does not require detergent for extraction and purification (Supplementary Information, Fig. 1d). The negative-stain EM of *Ct*pom152^186-1270^ also showed a beaded string structure (Fig. 1e) indistinguishable from that obtained for the chymotryptic fragment (*Ct*pom152^194-1256^) (Fig. 1c). Size-exclusion chromatography coupled to multi-angle light scattering (SEC-MALS) of *Ct*pom152^186-1270^ (at 6 mg/mL per injection) showed that it behaves as a monomer of 130.3 ± 9.6 kDa (Fig. 1f), close to its theoretical molar mass of 121.7 kDa. Likewise, SEC-MALS measurements carried out at two lower concentrations yielded similar molar masses (129.2 ± 11.2 kDa at 2 mg/mL or 136.5 ± 8.5 kDa at 4 mg/mL).

Our data so far left open the question of whether the *trans* regions of *Ct*pom152 could oligomerize into lumenal ring. We used purified *Ct*pom152^FL^, but removed much of the detergent (Supplementary Fig. S4) before rapidly freezing the sample for cryo-EM. As in the negative-stain images, we saw 44-nm long beaded strings (Fig. 1g, h). Some of these strings displayed sufficient density to unequivocally distinguish ten beads (Fig. 1i). Notably, other upstream elements of FL *Ct*pom152 were not visible in these cryo-electron micrographs, presumably because of their disordered structures; moreover, we could not detect the clear-cut density differences between the beads (Fig. 1i). We conclude that regions other than the *trans* domain of *Ct*pom152 are insufficiently compact to be visible in vitreous ice.

Strikingly, in up to an estimated 10% of images (over 2000 images), full-length *Ct*pom152 molecules appeared as much longer beaded structures (Fig. 1j), measuring in multiples of 44 nm without consistent punctuation marks between monomers (Fig. 1j). Because the *trans* segments lacking the N-terminal domain (*Ct*pom152^186-1270^ and *Ct*pom152^194-1256^) are unable to oligomerize, these data strongly suggested that the longer beaded structures might arise by head-to-tail oligomerization of the *trans* domains.

Intriguingly, both pre-Ig and Ig10 are amongst the most conserved regions between pom152 homologs (Supplementary Fig. S2), indicative of a common mechanism in yeast. To test it, we mapped the interacting sites between recombinant pre-Ig region and purified Ig10 (Fig. 1a, Supplementary Figs. S2 and S5) from both *Chaetomium thermophilum* and *Saccharomyces cerevisiae*. Indeed, both *Ct*pre-Ig and *Sc*pre-Ig immediately downstream from the TM sufficiently precipitated *Ct*Ig10 and *Sc*Ig10, respectively. The remarkable agreement thus strongly suggests that yeast pom152, independent of other poms (pom34 and Ndc1), is capable of assembling into a complete ring.

Taking together, our data here suggest that the large ‘*trans*’ domain of *Chaetomium thermophilum, Ct*pom152, an integral nuclear pore membrane protein, largely consists of *ten* closely linked Ig folds. Eight head-to-tail connected *trans* domains would assemble into a continuous ring with diameter of 100 nm^3,4^. Interestingly, our structural predictions of the presence of fifteen Ig folds in gp210 would yield correspondingly larger rings of 150 nm in diameter (Fig. 1k^5,6^ and Supplementary Fig. S3). Because of the symmetry, the 16 *trans* domains may form 2 eight-member rings, one situated above, the other below mid-plane, collectively representing the lumenal density around the waist of the pore membrane.

Why should NPC require such a structure? As the only conduit between nucleus and cytoplasm, dilation of the central channel to accommodate oversized particles could destabilize the pore membrane. Intriguingly, the *trans* domain of pom152 (or gp210) bears striking structural similarity to titin, an abundant protein in sarcomeres that fulfills a number of mechanical functions with the most notable one as a passive visco-elastic spring^7^. The existence of an elastic ring in lumen thus could provide the pore membrane with the counter force to maintain its integrity^5,8^.

While preparing our manuscript, a paper by Upla et al.^9^ was published, the results of which were both overlapping and divergent with those of our present paper. Based on 3D reconstruction of negative-stain EM, Upla et al.^9^ reported that full-length *Sc*pom152 consists of two distinct domains: (1) a large “head” group (comprising of its 375 N-terminal residues) (Fig. S1 and Fig. 4a of Upla et al.^9^), and (2) a “lumenal” domain consisting of nine beads in a rigid conformation, with the first one much longer than the remaining eight, all distinctly smaller than the head group (Fig. 6*a* of Upla et al.^9^). In contrast, our EM structures showed ten beads of about equal size in a highly flexible conformation (Fig. 1g, h, i), and the remaining fragment, terminating around the residue 214 (Fig. 1b and Supplementary Figs. S1 and S2), is insufficiently compact to be detected by cryoEM. Moreover, our biochemical analysis suggested that yeast pom152 can oligomerize in a conserved fashion: our predicted “head” (pre-Ig) of one *trans* domain directly interacts with the “tail” (Ig10) of another, whereby linking multi-*trans* domains into a single ring (Fig. 1, Supplementary Figs. S2 and S5). Surprisingly, unlike our model, the *Sc*pre-Ig region, *Sc*pom152^214-265^ was previously interpreted as part of the “head group” (Upla et al.^9^), not a constituent of the ring^3^. Better precision in the ‘head group’ boundary determination, negative stain in the absence of detergent, and higher resolution in 3D reconstruction will be needed in order to resolve the differences between our models.

## Electronic supplementary material

Supplementary Information

## Electronic supplementary material

**Supplementary Information** accompanies the paper at (10.1038/s41421-018-0057-7).
