# Supplementary material for: Electron microscopy of Chaetomium pom152 shows the assembly of ten-bead string
Source: Cell Discov. 2018 Sep 18;4:56. doi: 10.1038/s41421-018-0057-7 (PMC6141588; doi:10.1038/s41421-018-0057-7)
Supplement: Supplementary file 1 — Supplementary Information [file 41421_2018_57_MOESM1_ESM.docx]

**Electron microscopy of Chaetomium pom152 shows the assembly of ten-bead string**

Qi Hao^1,Δ^, Boyue Zhang^2^, Kangning Yuan^2^, Hang Shi^1, 2,*^  and Günter Blobel^1^

^1^Laboratory of Cell Biology, Howard Hughes Medical Institute, The Rockefeller University, 1230 York Ave., New York, NY 10065, USA; ^2^Beijing Advanced Innovation Center for Structural Biology, School of Life Sciences, Tsinghua University, Beijing 100084, China.

Running title: The Structure and *in vitro* Assembly of pom152

^*^To whom correspondence should be addressed:

Hang Shi

Office: (86)10 6277 3582

Email: hangshi@tsinghua.edu.cn

^Δ^current address: Calico Life Sciences, 1170 Veterans Blvd，South San Francisco, CA 94080, USA

Footnote: We would like to dedicate this article to Dr. Günter Blobel for his mentorship and unwavering pursuit of the nuclear pore complex. The initial discovery of the *trans* domain architecture and its oligomerization were made in Dr. Blobel’s laboratory at Rockefeller University. Dr. Blobel prepared the original manuscript. The remaining biochemical data and editing of the manuscript were carried out in Tsinghua University after he is deceased

**Supplementary information, Data S1**

*Cloning, Protein Expression and Purification -* DNA fragments encoding both full-length (FL) *Chaetomium thermophilum* (*Ct*) pom152 (*Ct*pom152^FL^) and the *trans* domain of *Ct*pom152^186-1270^ with C-terminal Histidine tag (8×His) were inserted between EcoRI and NotI sites of the pFastBac1 vector (Invitrogen). Bacmid DNAs were produced in DH10Bac cells, and baculoviruses were generated and subsequently amplified in Sf9 insect cells following standard protocols (Invitrogen). Both *Ct*pom152^FL^ and *Ct*pom152^186-1270^ were overexpressed in High Five (Hi5) insect cells (Invitrogen) grown in Sf-900TM II SFM medium (Invitrogen) at 27 ˚C with agitation at 110 rpm. Hi5 insect cells (1 L), with cell density at 1.0x10^6^ cells/mL, were infected with baculovirus (see Invitrogen standard protocol) and grown in suspension for 48 hours before harvest.

*Ct*pom152^FL^ expressing cells (1 L) were harvested and lysed on ice by sonication (Misonix sonicator 3000 with micro-tip) for 90 sec at 30% power in 40 mL buffer A [25 mM Tris-HCl, pH 8.0, 300 mM NaCl, 1 mM Phenylmethylsulfonyl fluoride (PMSF), and a quarter tablet of cOmplete™ EDTA-free Protease Inhibitor Cocktail (Roche)]. After centrifugation at 14,330×g for 30 min at 4 ˚C, the supernatant was incubated with Triton X-100 (2% final concentration) for 90 min at 4 °C. Following centrifugation at 38,360×g for 40 min at 4 ˚C, the supernatant was loaded onto a column containing 3 mL Ni^2+^-NTA resin (Qiagen). After washing the column with 35 mL buffer B [25 mM Tris pH 8.0, 300 mM NaCl, 35 mM imidazole pH 8.0, 0.2% Triton X-100], *Ct*pom152^FL^ was eluted with 15 mL buffer C [25 mM Tris pH 8.0, 300 mM NaCl, 350 mM imidazole pH 8.0, 0.2% Triton X-100], and, after concentration, was purified by Superdex 200 (10/300) size-exclusion chromatography (GE Healthcare) using buffer D [25 mM Tris pH 8.0, 150 mM NaCl, 0.02% Triton X-100]. Purified protein was concentrated and stored at -80 °C at 0.8 mg/mL. To reduce the detergent for MALS and cryo-EM, 7 mL purified protein was further incubated with 1 mL pre-treated Bio-Beads^TM^-2 Adsorbent (Cat. #152-8920, Bio-rad) for 12 h at 4 ˚C with constant agitation.

Identical lysis procedures were used for *Ct*pom152^186-1270^, and the lysate was cleared by centrifugation at 38,360×g for 45 min at 4 ˚C. The supernatant was first purified by Ni^2+^-NTA affinity chromatography following the same procedure as that of the full-length protein except that Triton X-100 was excluded from all buffers. *Ct*pom152^186-1270^ was further purified by anion-exchange chromatography using a 5 mL HiTrap Q HP column (GE Healthcare) and a gradient of 0 to 600 mM NaCl in 25 mM Tris-HCl, pH 8.0. The eluted protein was concentrated and subsequently purified by Superdex 200 (10/300) size-exclusion chromatography using buffer E (25 mM Tris-HCl, pH 8.0 and 150 mM NaCl), the peak fractions were concentrated to 6mg/mL and stored at -80 °C.

DNA fragments encoding pre-Ig domain (*C*tpom152^164-214^, *Ct*pom152^164-303^ and *Sc*pom152^214-265^) and Ig10 (including *Ct*Ig10^1154-1270^ and *Sc*Ig10^1226-1337^) were inserted to the modified pGEX-KG vector. *E. coli* strain BL21 (DE3) harboring plasmids expressing Ig10 (*Sc*Ig10^1226-1337^ and *Ct*Ig10^1154-1270^) were grown in 2 L LB containing 100ug/mL Ampicillin at 37 ˚C. IPTG was added at final concentration of 1 mM when the cell density reached OD_600_ =0.6~0.8. The cells were allowed to grow for another 16 hours at 20 ˚C, harvested by centrifugation at 4000 rpm for 20 min at 4 ˚C and then washed once with 1x PBS (137 mM NaCl, 2.7 mM KCl, 10 mM Na_2_HPO_4_, 1.8 mM KH_2_PO_4_ pH7.2). After harvesting, cells were lysed at 4 ˚C by high pressure cell disruptor (UH-06，Union Biotech). Cell lysate was cleared by centrifugation at 14000 rpm for 30 min at 4 ˚C and the supernatant was fist loaded onto a column containing 10 mL Glutathione Sepharose^TM^ 4 Fast Flow affinity resin (GE Healthcare) that was previously balanced with 50 ml lysis buffer. After washing, Ig10 was released by incubating with 30 U thrombin in 6 mL digestion buffer (200 mM NaCl, 10 mM Hepes pH 8.0, 1 mM DTT) for 16 hours at 25 ˚C. The eluted *Sc*Ig10^1226-1337^ was further purified by anion-exchange chromatography using a 5 mL HiTrap Q HP column (GE Healthcare) and a NaCl gradient (from 50 mM to 1 M) in 10 mM Hepes, pH 8.0 and 1 mM DTT; whereas the eluted *Ct*Ig10^1154-1270^ was further purified by cation-exchange chromatography using 5 mL HiTrap SP column (GE Healthcare) using the identical buffers for gradient. The purified protein was concentrated using ultracel-3K centrifugal filter (Amicon Ultra-4) to 5.3 mg/mL.

*Limited Proteolysis assay - Ct*pom152^FL^ (10 μL at 0.8 mg/mL) was mixed with chymotrypsin (10 μL at 20 μg/mL in buffer D) and incubated at 25 °C for 15 min. The reaction was stopped by the addition of PMSF at a final concentration of 1 mM, and protein samples were subjected to SDS-PAGE and visualized by Coomassie Blue staining. Mass spectrometry analyses of an excised gel slice containing the largest digestion product (indicated in Fig. 1**b**) showed it included amino acids 194 to 1256; this large chymotryptic fragment was therefore termed *Ct*pom152^194-1256^.

To generate larger quantities of *Ct*pom152^194-1256^, 1 mL *Ct*pom152^FL^ (2 mg/mL) was incubated with 50 μg/mL chymotrypsin (Sigma-Aldrich Cat No. C4129) at 25 °C for 15 min. The reaction was stopped by the addition of PMSF at a final concentration of 1 mM. The resulting fragment was further purified by Superdex 200 (10/300) size-exclusion chromatography using buffer E and then concentrated and stored at -80 °C at 2 mg/mL.

*Negative-Stain EM -* Carbon-coated copper grids (CF200-Cu, 200 mesh, Electron Microscopy Sciences) were glow-discharged for 45 sec. The grids were then loaded with 5 μL of *Ct*pom152^194-1256^ (10 μg/mL, Fig. 1c) or *Ct*pom152^186-1270^  (2 μg/mL, Fig. 1e) in their storage buffer and incubated for 2 min. Each grid was first washed twice with 30 μL of Milli-Q water, and then washed once and incubated once with 25 μL of freshly prepared 1% uranyl acetate for 2 min. Images were acquired using a FEI TECNAI G2 Spirit BioTwin Transmission Electron Microscope (120 kv) equipped with a Gatan 4K × 4K digital camera with the defocus value set between -1 and -3 μm.

*Cryo-EM –* Holy carbon grid (CF-2/2-4C-T, Protochips) was glow discharged for 20 sec. The grid was loaded with 5 µL *Ct*pom152^FL^ (0.6 mg/mL), immediately blotted with filter paper for five sec at 22 °C and 100% humidity, and plunge frozen in liquid ethane using a vitrobot (FEI Vitrobot Mark VI). Images were collected using FEI Talos Arctica electron microscope (200 kV) equiped with a direct electron detector (Gatan K2 summit) at 28,000× magnification with defocus between -3 and -4 µm.

*Mass Spectrometry* - Coomassie Blue stained proteins in the excised gel band were reduced in *situ* with DTT and alkylated with iodoacetamide, followed by overnight trypsinization; tryptic peptides were solubilized from the gel, dried down and then solubilized in 20 μL 5% acetonitrile/0.1% formic acid, and a portion was analyzed by reversed phase nano LC-MS/MS (Q-Exactive mass spectrometer/Dionex LC, Thermo Fisher, San Jose, CA, USA). Tandem MS data was extracted using Proteome Discoverer v. 1.4 (Thermo Fisher, Bremen, Germany) and queried (Matrix science, London, UK) against the Uniprot *Chaetomium thermophilum* database. *Ct*pom152 was matched at a peptide false discovery rate of 1% or better.

*Multi-Angle Light Scattering (MALS) -* One hundred (100) μL of *Ct*pom152^186-1270^ (2 mg/mL, 4 mg/mL or 6 mg/mL in 25 mM Tris-HCl, pH 8.0, 150 mM NaCl was applied to a Superdex 200 Increase 10/300 GL column (GE Healthcare) connected in series with an 18-angle light scattering detector (DAWN HELEOS) and refractive index detector (Optilab T-rEX; Wyatt Technology). Data was collected every 1 sec with a flow rate of 0.15 mL/min at 25 ˚C. For *Ct*pom152^FL^, one hundred (100) μL *of* 0.5mg/mL detergent removed protein was applied to a Protein KW-803 column (Shodex) using same buffer and MALS system as that for *Ct*pom152^186-1270^. Data was collected every 1 sec with a flow rate of 1 mL/min at 25 ˚C. All data was processed using ASTRA (Wyatt Technology). The molar mass of the minor peak eluted before 10 ml corresponds to protein aggregates that measure over 3000 kDa which showed no defined structure when viewed by negative-stain EM.

*Pull-down analysis* - BL21 (DE3) containing empty vector (pGEX-KG, for GST alone) or plasmids (*Ct*pom152^164-214^, *Ct*pom152^164-303^ or *Sc*pom152^214-265^) was grown in 100 mL LB containing Ampicillin (100 ug/mL) at 37 ˚C. IPTG was added at final concentration of 1 mM when the cell density reached OD_600_ =0.6~0.8. The cells continued to grow for another 16 hours at 20 ˚C. *E. coli* was harvested after centrifugation at 4000 rpm for 20 min at 4 ˚C then washed once with 1xPBS (137 mM NaCl, 2.7 mM KCl, 10 mM Na_2_HPO_4_, 1.8 mM KH_2_PO_4_ pH7.2). The proteins were lysed on ice by sonication (JY92-IIN, SCIENTZ) for 15 min at 24% power in 3 mL lysis buffer [10 mM Hepes pH 8.0, 1 M NaCl, 1 mM DTT with one tablet of cOmplete™ EDTA-free Protease Inhibitor Cocktail (Roche) per 200 mL buffer]. 2.8 mL supernatant were recovered after centrifugation at 14000 rpm for 30 min at 4 ˚C.

The supernatant containing GST fusion pre-Ig domains (1 mL) was incubated with 100 uL pre-equalibrated Glutathione Sepharose^TM^ 4 Fast Flow resin (GE Healthcare) for 30 min at 4 ˚C with gentle agitation then pelleted by centrifugation at 700 rpm for 5 min at 4 ˚C. After washing twice with 1 mL lysis buffer, the resin was incubated with 20 uL Ig10 (*Sc*Ig10^1226-1337^ or *Ct*Ig10^1154-1270^) in 1 mL binding buffer (150 mM NaCl, 10 mM Hepes pH 8.0) at 4 ˚C over night with constant agitation. Afterwards, the resins were washed by 1mL lysis buffer twice. All samples were analyzed using tricine gel followed by coommassie blue staining (Supplementary Fig. S5).

*Structure Prediction* **-** The full-length amino acid sequences of both *Ct*pom152 (Gene ID: 18258921) and *Hs*gp210 (Gene ID: 116242720) were submitted to the Protein Fold Recognition server, Phyre2 ^10^, to retrieve homology folds. The boundaries and the secondary structures of the predicted domains were assigned according to alignment to the prediction templates with the highest confidence score. The secondary structures for regions without domain assignment were determined using the PredictProtein server ^11^. Secondary structures, domain boundaries, template coordinates (protein data bank code), and confidence scores are shown in supplementary Fig. S2 and S3. The sequence alignment was calculated by ClustalW^12^ and prepared by Jalview^13^.

**REFERENCES:**

10 Kelley, L. A., Mezulis, S., Yates, C. M., Wass, M. N. & Sternberg, M. J. The Phyre2 web portal for protein modeling, prediction and analysis. *Nature protocols* **10**, 845-858, doi:10.1038/nprot.2015.053 (2015).

11 Yachdav, G. *et al.* PredictProtein--an open resource for online prediction of protein structural and functional features. *Nucleic acids research* **42**, W337-343, doi:10.1093/nar/gku366 (2014).

12 Thompson, J. D., Gibson, T. J. & Higgins, D. G. Multiple sequence alignment using ClustalW and ClustalX. *Current protocols in bioinformatics / editoral board, Andreas D. Baxevanis ... [et al.]* **Chapter 2**, Unit 2 3, doi:10.1002/0471250953.bi0203s00 (2002).

13 Waterhouse, A. M., Procter, J. B., Martin, D. M., Clamp, M. & Barton, G. J. Jalview Version 2--a multiple sequence alignment editor and analysis workbench. *Bioinformatics* **25**, 1189-1191, doi:10.1093/bioinformatics/btp033 (2009).

14 Wozniak, R. W., Blobel, G. & Rout, M. P. POM152 is an integral protein of the pore membrane domain of the yeast nuclear envelope. *The Journal of cell biology* **125**, 31-42 (1994).

15 Tcheperegine, S. E., Marelli, M. & Wozniak, R. W. Topology and functional domains of the yeast pore membrane protein Pom152p. *The Journal of biological chemistry* **274**, 5252-5258 (1999).

16 Lau, C. K., Delmar, V. A. & Forbes, D. J. Topology of yeast Ndc1p: predictions for the human NDC1/NET3 homologue. *The anatomical record. Part A, Discoveries in molecular, cellular, and evolutionary biology* **288**, 681-694, doi:10.1002/ar.a.20335 (2006).

17 Miao, M., Ryan, K. J. & Wente, S. R. The integral membrane protein Pom34p functionally links nucleoporin subcomplexes. *Genetics* **172**, 1441-1457, doi:10.1534/genetics.105.052068 (2006).

18 Natarajan, K. M., Michael G; and Margulies, David H. Immunoglobulin Superfamily. *eLS*, 7, doi:10.1002/9780470015902.a0000926.pub2 (2015).

**Supplementary Figure S1
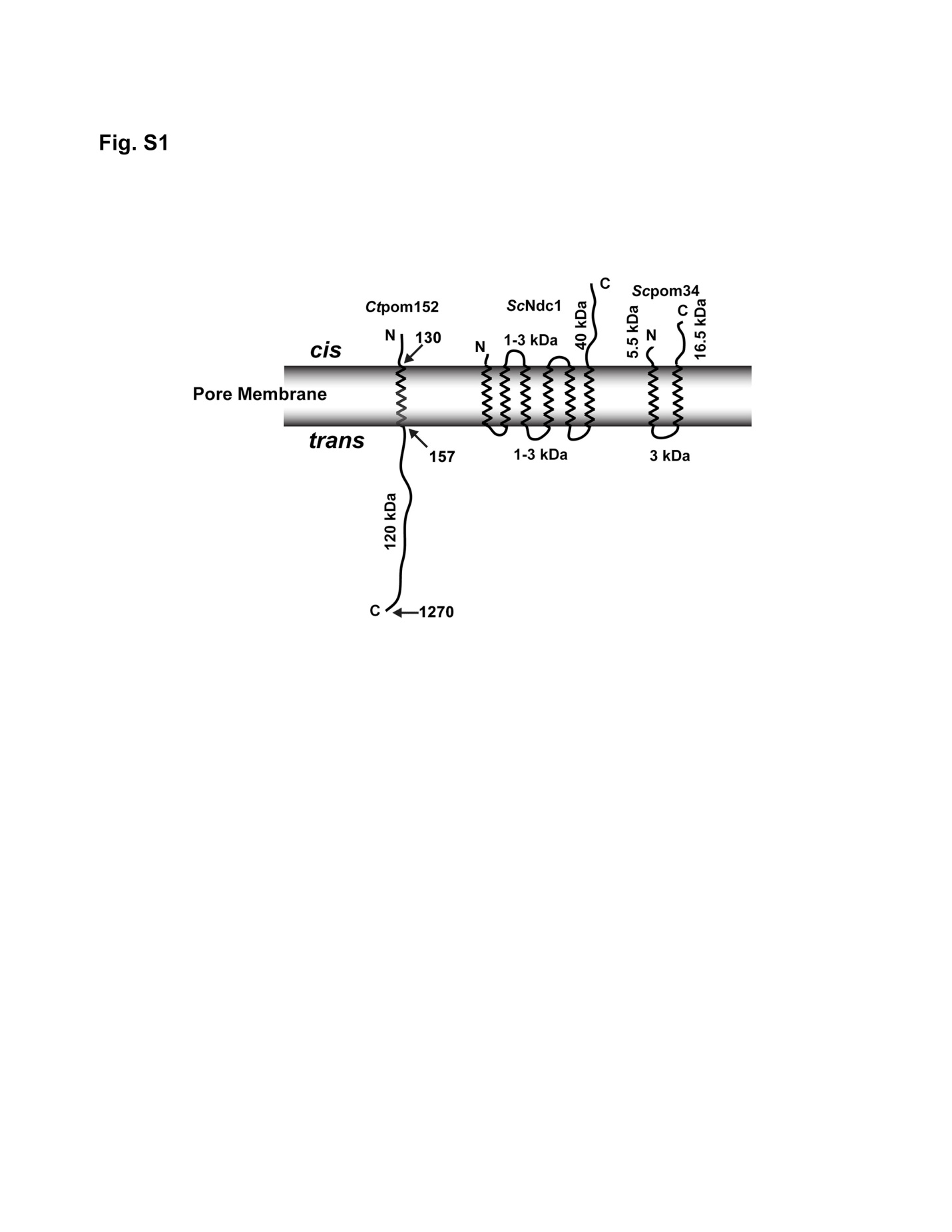
Supplementary Figure S1** Overview of the topology of the three protein constituents of the nuclear pore membrane of yeasts, *Ct*pom152 ^14,15^, *Sc*Ndc1 ^16^ and *Sc*pom34 ^17^, and their molar masses (in kDa); ‘*cis*’ and ‘*trans*’ refer to cytoplasmic and biosynthetically-translocated domains, respectively; arrows indicate relevant residue numbers on *Ct*pom152.

**
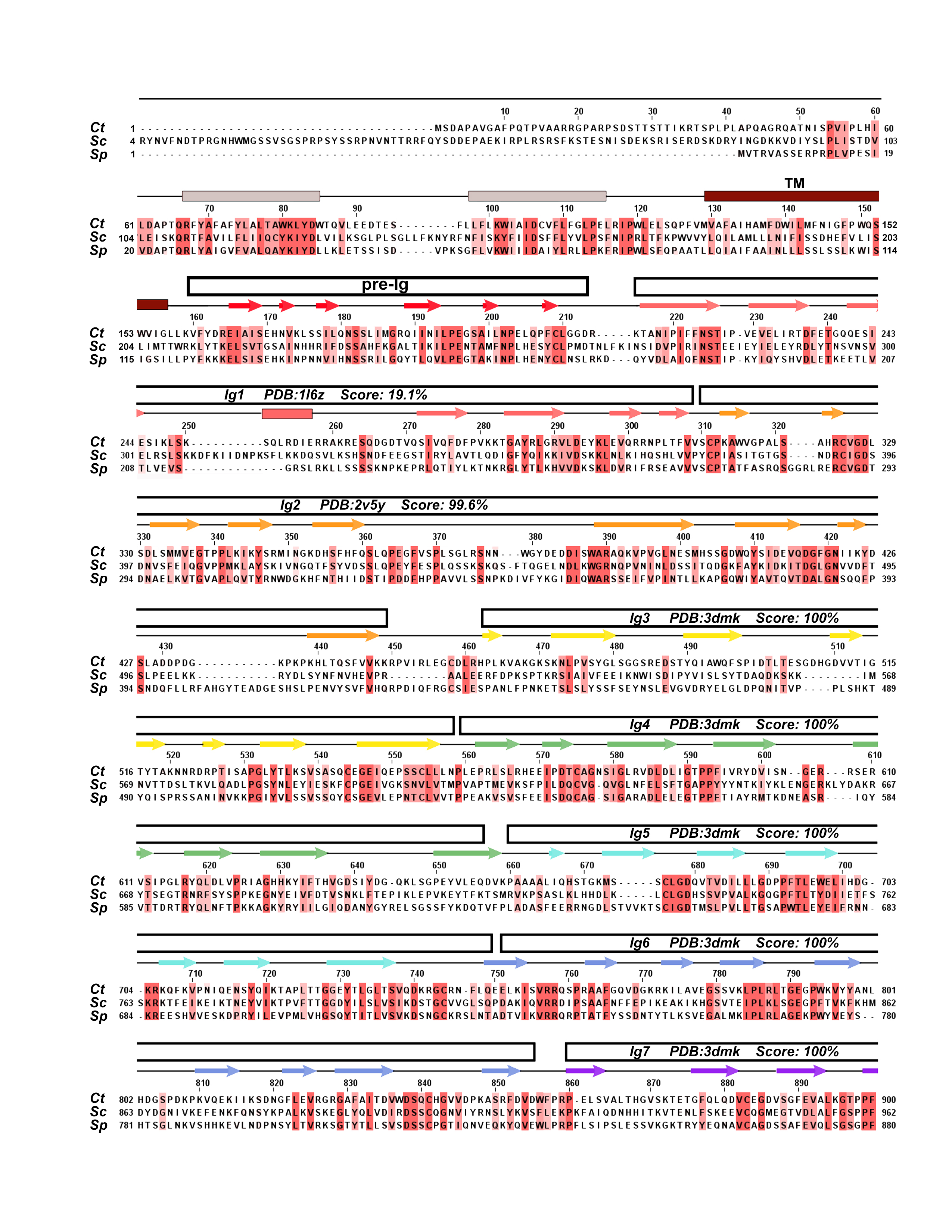
Supplementary Figure S2**

**
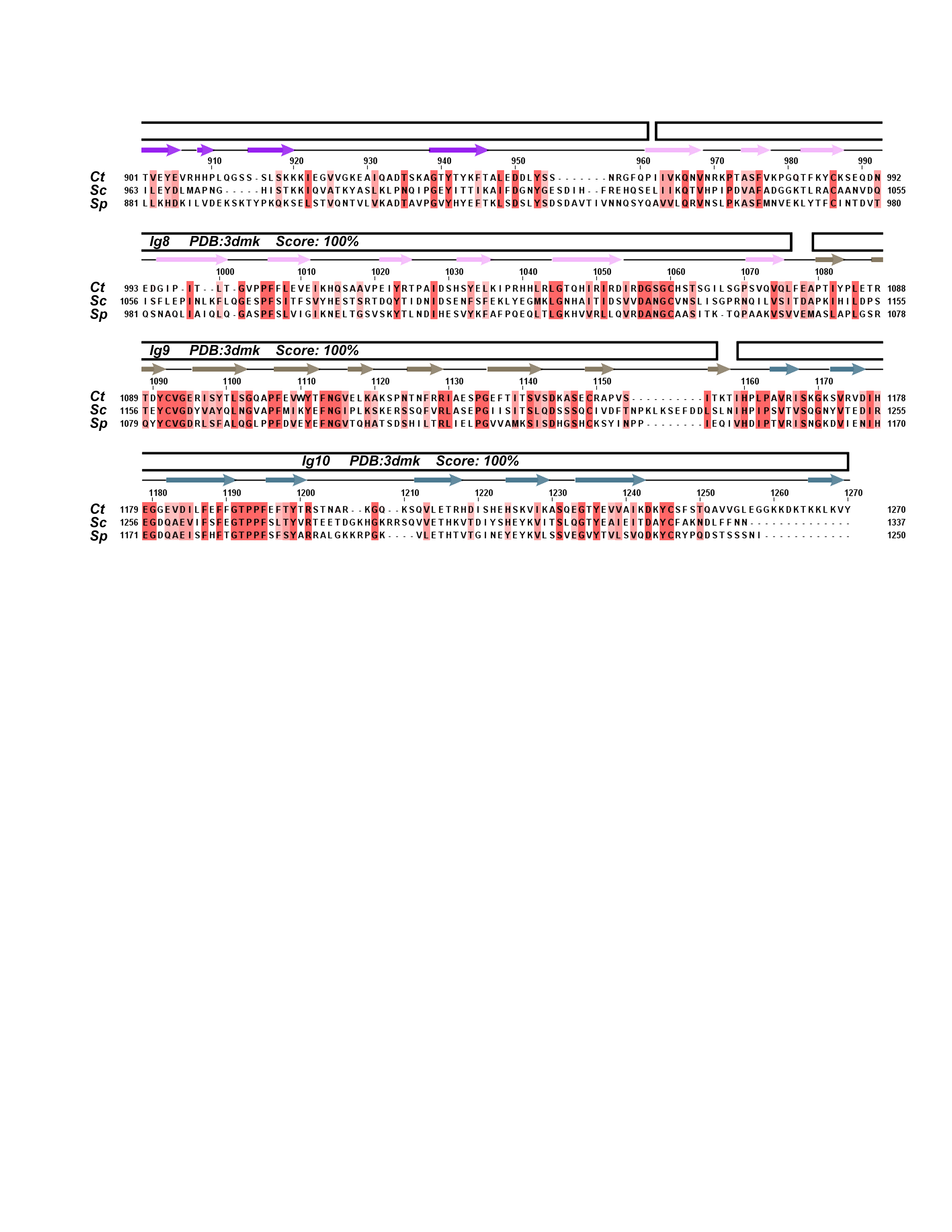
**

**Supplementary Figure S2. The *trans* domain of fungal pom152 contains ten Ig-like domains.** The amino acid sequence, secondary structure and fold of *Chaetomium thermophilum,* *Ct*pom152 were predicted with Phyre2 and PredictProtein servers. Sequence alignment to *Saccharomyces cerevisiae* (*Sc*) and *Schizosaccharomyces pombe* (*Sp*) was carried out with ClustalW ^12^ and presented using Jalview ^13^ [colored according to conservation from white (0%) to red (100%)]. The ten consecutive Ig-like domains (designated Ig1 through Ig10, see also Fig. 1a) are indicated by black boxes with domain name, model templates PDB code, and prediction confidence score. Each Ig-like fold contains an average of seven β strands (arrows; color coded according to the domain) interrupted by loops (solid lines) and helices (solid rectangles) ^18^. One transmembrane helix (TM, dark red rectangle) is predicted near the amino terminus of each yeast pom152 which is followed by a highly conserved pre-Ig region (black box with domain name, also see Fig. 1a). Two additional hydrophobic helices (grey rectangle), located N-terminal to the transmembrane helix, might be interacting sites for nucleoporins.

**
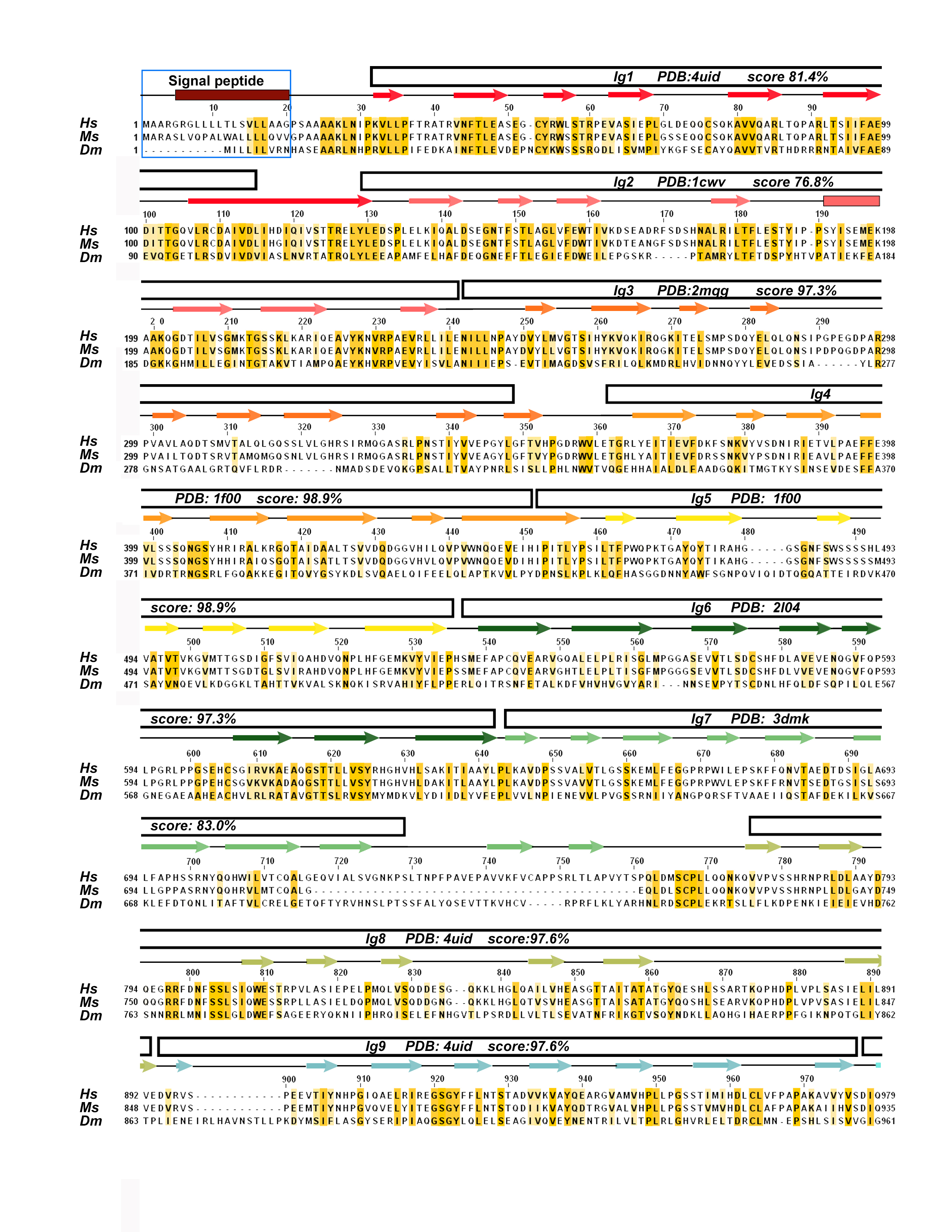
Supplementary Figure S3**

**
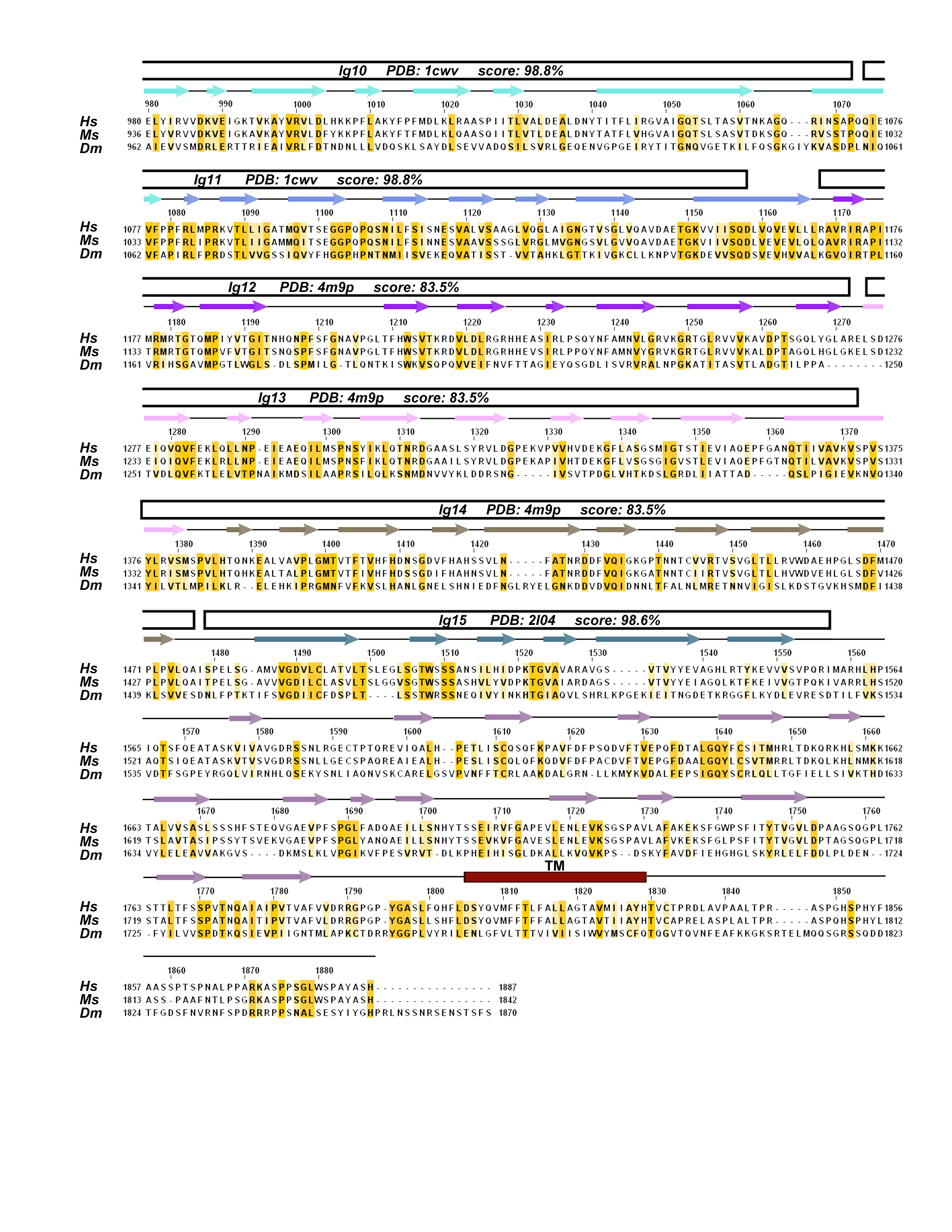
**

**Supplementary Figure S3. The *trans* domain of metazoan gp210 contains 15 Ig-like domains.** The amino acid sequence, secondary structure and fold of *Homo sapiens* (*Hs*) gp210 were predicted with Phyre2 ^10^ and predictprotein servers ^11^. The sequence was aligned with homologs from *Mus musculus* (*Ms*) and *Drosophila melanogaster* (*Dm*) using ClustalW ^12^ and presented using Jalview ^13^ [colored according to conservation from white (0%) to orange (100%)]. *Hs*gp210 primarily contains β strands that organize into fifteen consecutive Ig-like domains (Ig1 through Ig15, Fig. 1a, black box with domain number, model template PDB code and prediction confidence score). Each Ig-like domain contains an average of seven β strands (arrows, color coded according to the domains) interrupted by loops (solid lines)^18^. One amino-terminal helix (dark red rectangle) is part of the signal peptide (blue box) and another C-terminal helix functions as a transmembrane segment (TM, dark red rectangle).

**
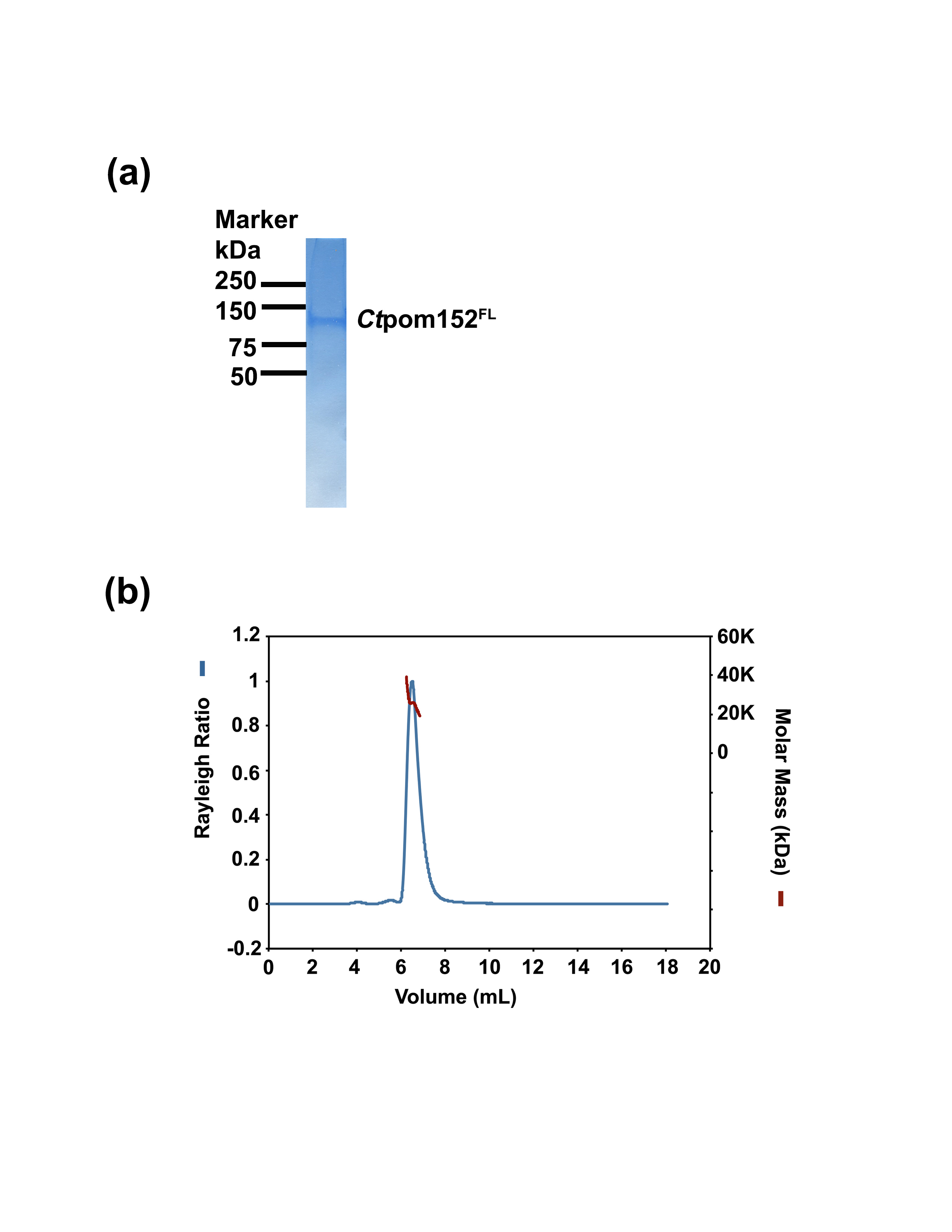
Supplementary Figure S4­**

a

**Supplementary Figure S4. Purification of *Ct*pom152^FL^. a**, *Ct*pom152^FL^ used for cryoEM analysis was first affinity purified by Ni^2+^-NTA resin, pre-incubated with biobeads to remove detergent and then subject to size-exclusion chromotography (see experimental procedures) to achieve high purity. **b**, Size exclusion chromatography coupled to multiple angle light scattering (SEC-MALS) indicates that *Ct*pom152^FL^ form high molecular oligomer that measures 26.6±0.1 MDa when detergent is depleted.

b

**Supplementary Figure S5**


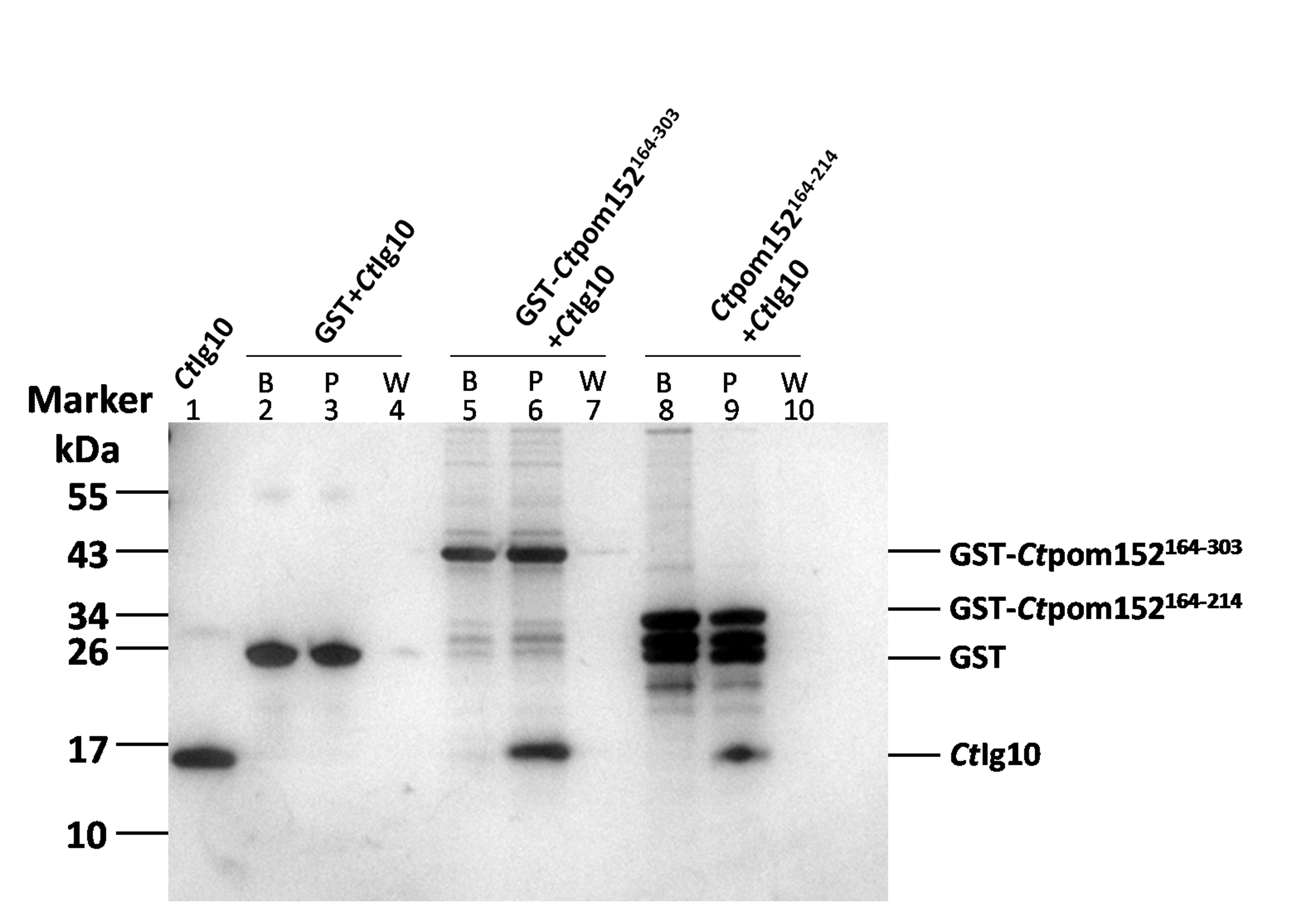


**a**


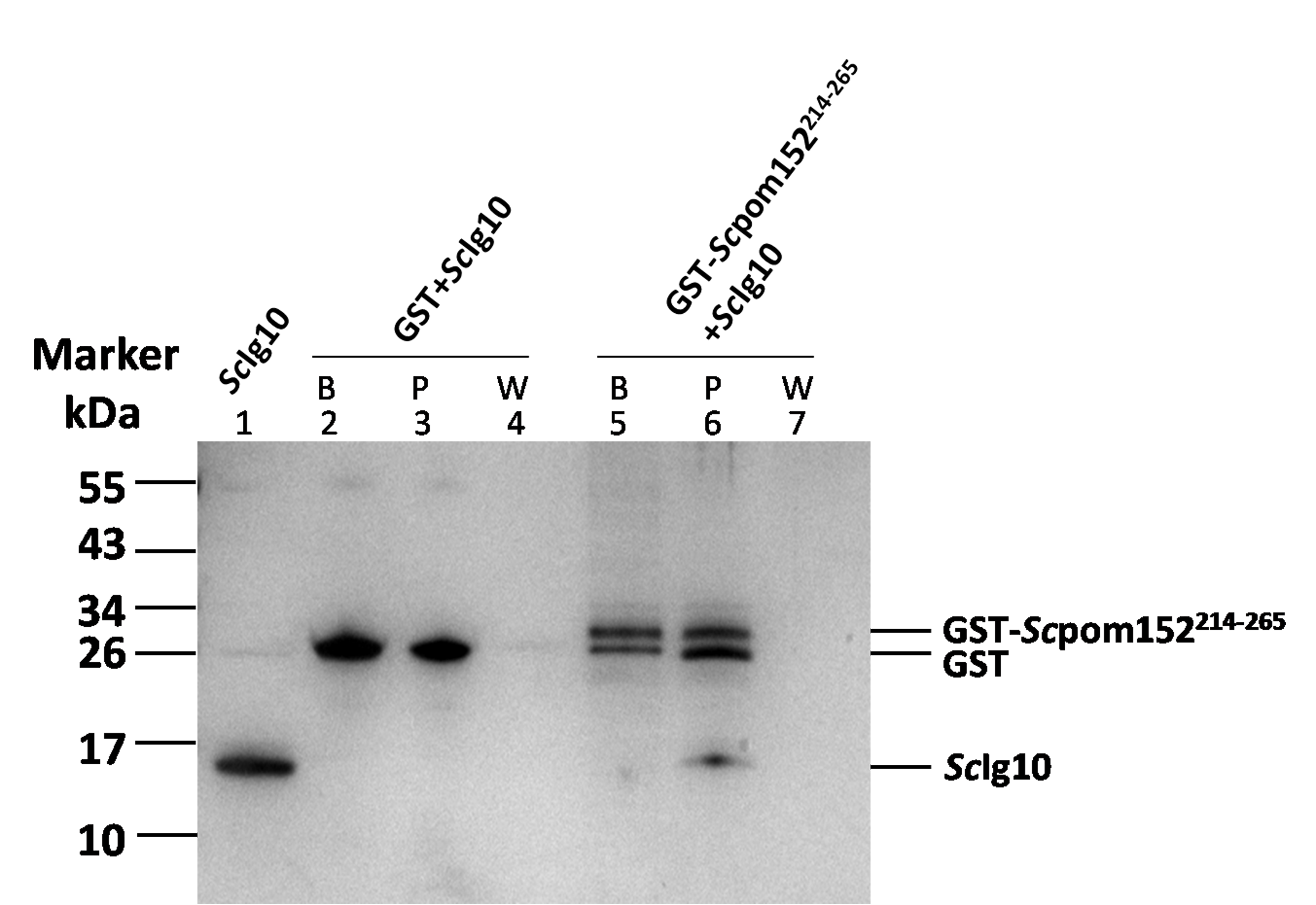


**b**

**Supplementary Figure S5. Ig10 interacts directly with pre-Ig region in both *Chaetomium thermophilum* and *Saccharomyces cerevisiae*.** **a**, GST (lane 2-4), GST-*Ct*pom152^164-303^ (lane 5-7) and GST-*Ct*pom152^164-214^ (lane 8-10) were first immobilized on glutathione sepharose beads (lane 2, 5 and 8) then incubated with purified *Ct*Ig10 (*Ct*Ig10^1154-1270^, lane 1). **b**, Similar to a, purified *Sc*Ig10^1226-1337^ (Lane 1) was incubated with immobilized GST (lane 2-4) and GST-*Sc*pom152^214-265^ (lane 5-7), respectively. Beads with immobilized GST or GST-pre-Ig regions (as bait, B), beads after pull-down Ig10 (P), buffer after wash (W) were analyzed by Tricine gel and stained with Coomassie blue.
